# Supplementary material for: Single-Cell Sequencing Reveals the Heterogeneity of Hepatic Natural Killer Cells and Identifies the Cytotoxic Natural Killer Subset in Schistosomiasis Mice
Source: Int J Mol Sci. 2025 Mar 30;26(7):3211. doi: 10.3390/ijms26073211 (PMC11989782; doi:10.3390/ijms26073211)
Supplement: Supplementary file 1 [file ijms-26-03211-s001.zip › ijms-3462847-supplementary.pdf]

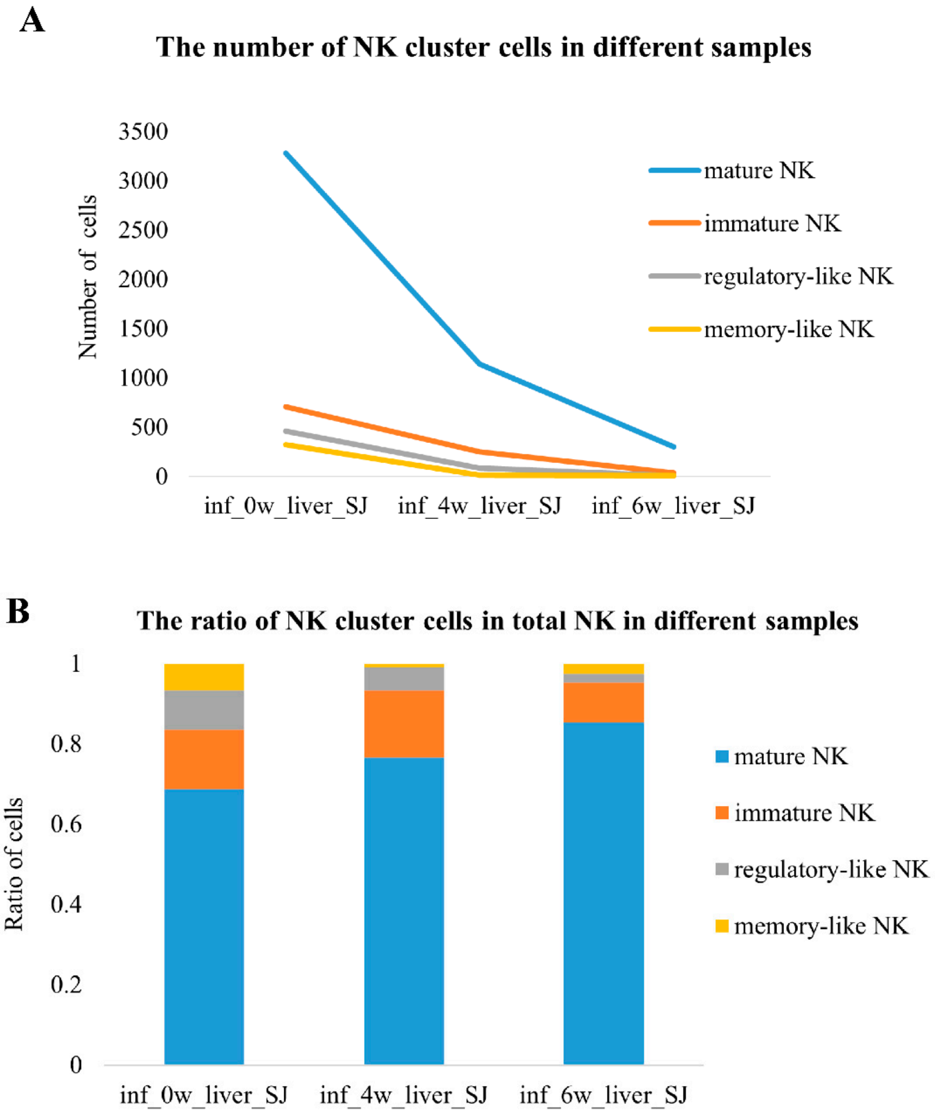

Supplementary Figure S1. The number and ratios of NK clusters in different samples. (A) Number of NK clusters in different samples. (B) Ratios of NK clusters in total NK in different samples.

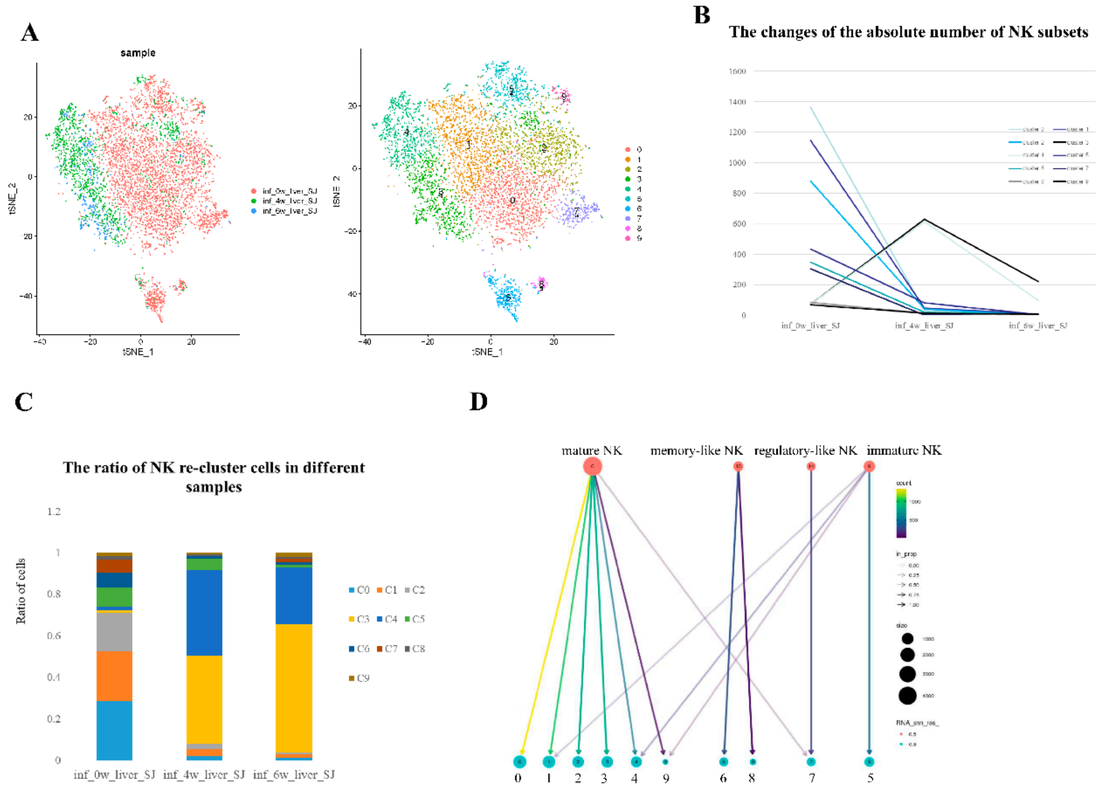

Supplementary Figure S2. Re-clustering of NK cells. (A) Visualizing single-cell data using tSNE. The data show the annotation and color codes for cell types in enrichment hepatic NK cells. The numbers 0-9 refer to C0 toC9. (B) Changes in the absolute number of 10 NK cell sub-clusters after *S. japonicum* infection. (C) Ratios of NK cell subsets in different samples. (D)The correspondence between four clusters of NK cells and 10 sub-clusters.

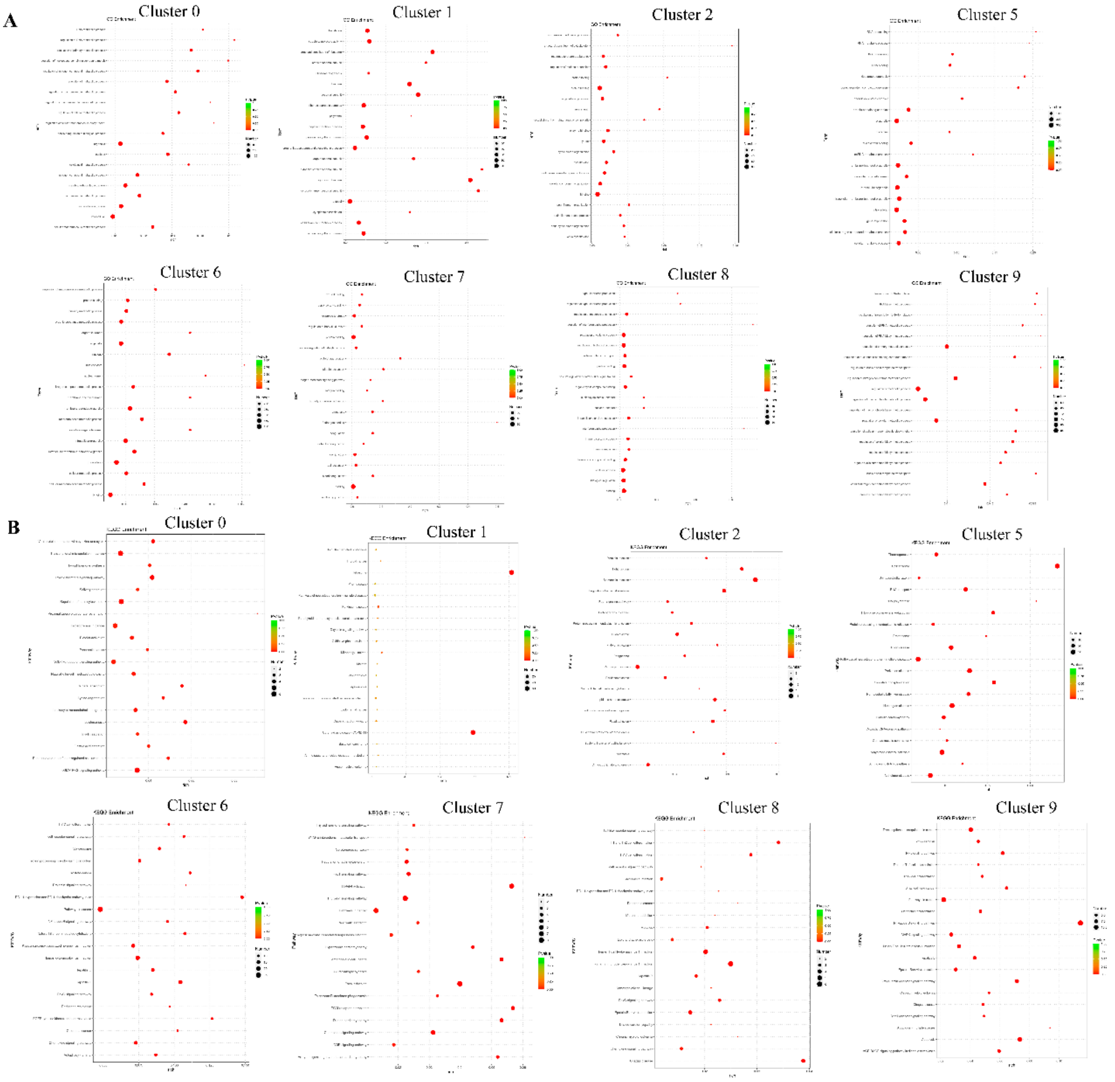

Supplementary Figure S3. GO and KEGG enrichment analysis of the remaining 8 clusters except for C3 and C4. (A) Go enrichment analysis of the remaining 8 clusters except for C3 and C4. (B) KEGG enrichment analysis of the remaining 8 clusters except for C3 and C4.

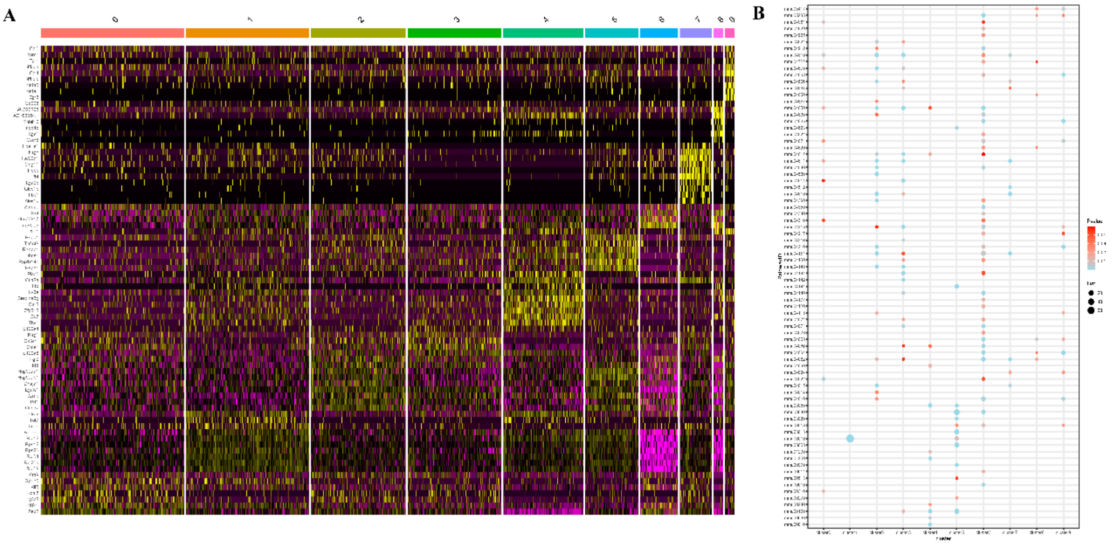

Supplementary Figure S4. The characteristics of 10 NK cell sub-clusters. (A) Gene expression in each NK cell sub-cluster. (B) 79 signal pathways activated in NK cells.

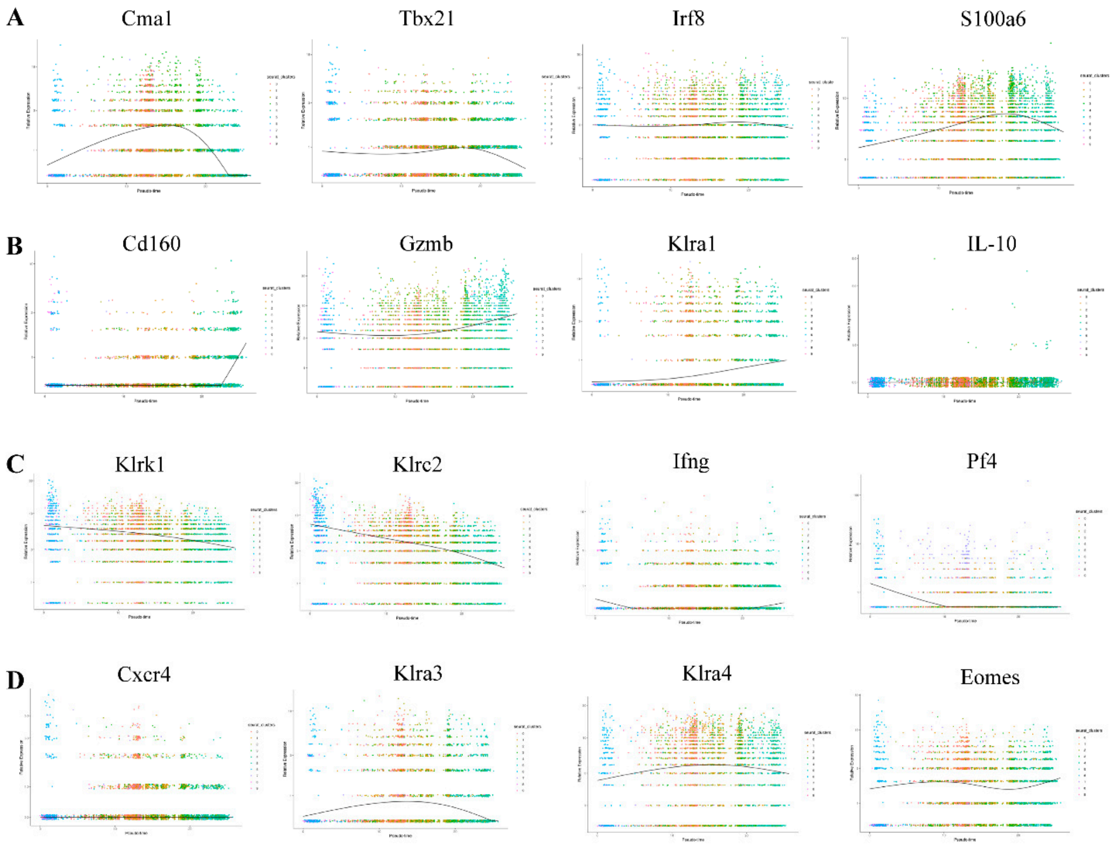

Supplementary Figure S5. Pseudo-time analysis of gene expression in four NK clusters. (A) Pseudo-time analysis of genes in mature NK cells. (B) Pseudo-time analysis of genes in immature NK cells. (C) Pseudo-time analysis of genes in memory-like NK cells. (D) Pseudo-time analysis of genes in regulatory-like NK cells.

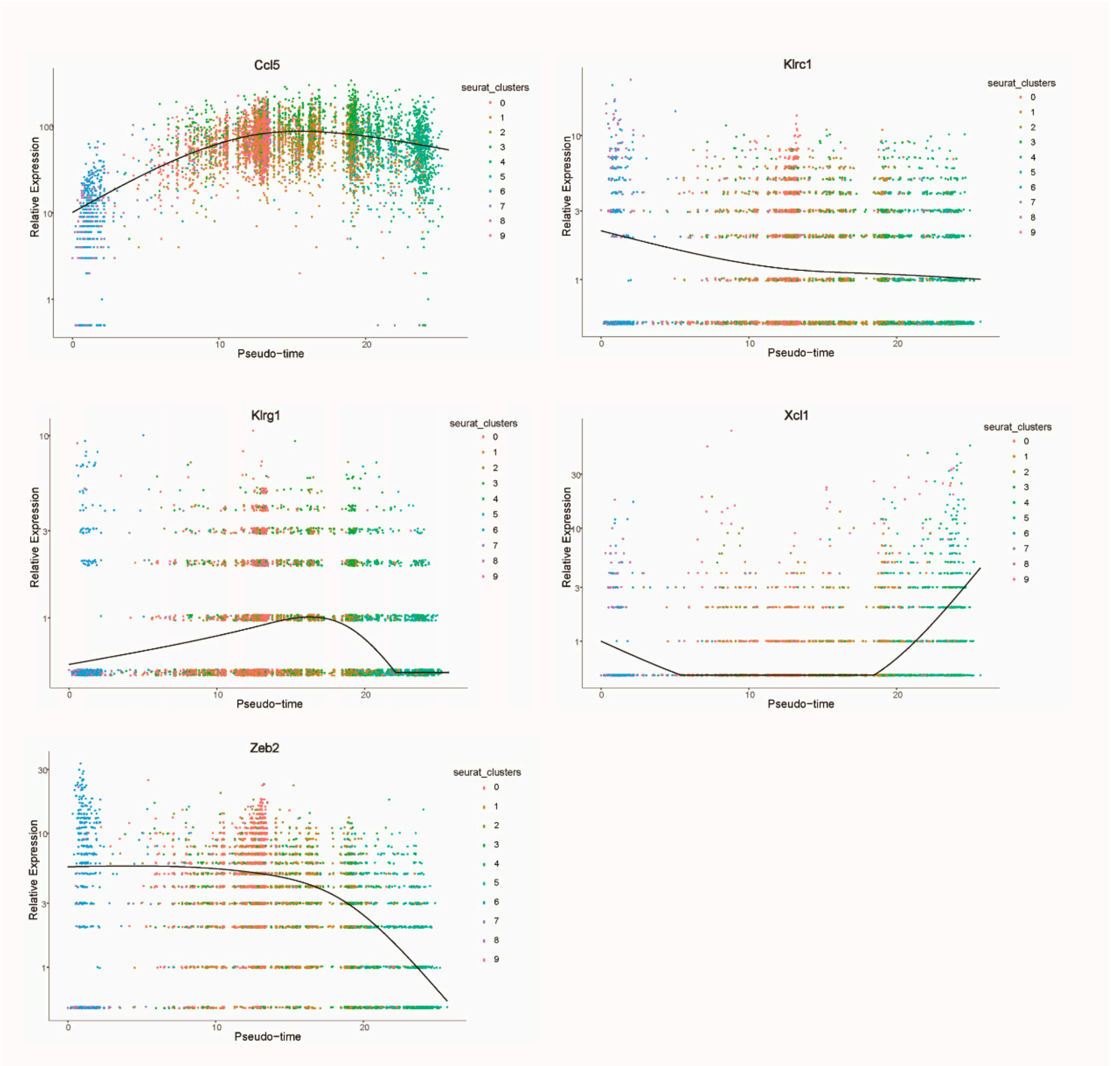

Supplementary Figure S6. Trajectory analysis of specific genes.

Supplementary Table S1. Primer sequences for qPCR of marker genes in each NK cell cluster.

Gene name	Forward primer sequence (5'-3')	Reverse primer sequence (5'-3')
**β-actin**	CATTGCTGACAGGATGCAGAAGG	TGCTGGAAGGTGGACAGTGAGG
**Kcnj8**	CTGTTGATAATCCCATCGAGAGC	CTCGAGAATCACTATGACCTCCAG
**Cmah**	GAAACAGATGAAGATTTCAGCCC	CGGAAAGGATAAATCTAGAAAGTCC
**Ccnd**	GCAGAAGGACATCCAACCGTAC	ACTCCAGCCAAGAAACGGTCCA
**Cx3cr1**	GAGCATCACTGACATCTACCTCC	AGAAGGCAGTCGTGAGCTTGCA
**Thy1**	CCTTACCCTAGCCAACTTCACC	TTATGCCGCCACACTTGACCAG
**Tnfrsf9**	CCAAGTACCTTCTCCAGCATAGG	GCGTTGTGGGTAGAGGAGCAAA
**Zbtb20**	CCTCATCCACTCGACACATTCAC	GAAGGTTGATGCTGTGAATGCGC
**Gng11**	ACTGAAGATGGAGGTTGAGCAA	TTCAATGTAGTTCTTTATTTCCTCAG
**CD226**	CACACTCACTTGCCAGCTTCC	TCTCTTGAGATAGGTTACAGGAAGC
**Egr3**	TGACCAACGAGAAGCCCAATC	CCAGTTGGAAGGAGAGTCGAAAG

Supplementary Table S2. Primer sequences for qPCR of Gzmb and Prf1 in NK92 cells.

Gene name	Forward primer sequence (5'-3')	Reverse primer sequence (5'-3')
**β-actin**	CACCATTGGCAATGAGCGGTTC	AGGTCTTTGCGGATGTCCACGT
**Thy1**	GAAGGTCCTCTACTTATCCGCC	TGATGCCCTCACACTTGACCAG
**Gzmb**	CGACAGTACCATTGAGTTGTGCG	TTCGTCCATAGGAGACAATGCCC
**Prf1**	ACTCACAGGCAGCCAACTTTGC	CTCTTGAAGTCAGGGTGCAGCG
